# Fifteen coronary angiograms, nine stents, and surgical denervation: unresolved vasospastic angina in a young patient—a case report

**DOI:** 10.3389/fcvm.2026.1759861

**Published:** 2026-02-02

**Authors:** Olivier Gach, Arthur Dumont, Claudiu Ungureanu, Lucien Finianos, Pieter-Jan Palmers, Giuseppe Colletti, Timothée Noterdaeme

**Affiliations:** 1 Cardiovascular Department, MontLégia, Liège, Belgium; 2 UC Louvain, Clinique Universitaire Saint-Luc, Bruxelles, Belgium; 3 Cardiovascular Department, Jolimont Hospital, La Louvière, Belgium; 4 Cardiovascular Department, Clinique Saint-Joseph, Arlon, Belgium

**Keywords:** brain–heart interaction, cardiac denervation, overtreatment, percutaneous coronary intervention, refractory angina, vasospastic angina

## Abstract

**Introduction:**

Vasospastic angina (VSA) can mimic obstructive coronary disease and may lead to overtreatment.

**Case presentation:**

A middle-aged Caucasian man with recurrent chest pain underwent stenting and escalating therapy despite negative angiograms. Subsequent provocative testing confirmed a diagnosis of refractory VSA. Although autonomic modulation improved symptoms, recurrence of symptoms suggested persistent endothelial dysfunction and complex pain mechanisms.

**Conclusion:**

This case underscores the importance of early functional testing to prevent unnecessary interventions and support a tailored, holistic approach to the management of VSA.

## Highlights

- Refractory vasospastic angina may persist despite complete autonomic cardiac denervation, highlighting the role of non-neuronal mechanisms, such as endothelial dysfunction.
- In patients with recurrent chest pain and patent stents, coronary vasospasm should be considered early, and functional coronary testing should be prioritized.
- Procedural overuse in clinically ambiguous scenarios can worsen endothelial dysfunction and increase psychological distress.
- Persistent angina following denervation may involve central nervous system processing of pain, emphasizing the need to integrate neuropsychological assessment into cardiovascular care.

## Introduction

Vasospastic angina (VSA) represents a diagnostic and therapeutic challenge for clinicians, particularly when it coexists with atherosclerotic coronary artery disease (1, 2). Characterized by transient vasoconstriction of the epicardial or microvascular coronary arteries, VSA can mimic acute coronary syndromes (ACS) and is often difficult to distinguish from ischemia caused by fixed coronary stenosis (3). In daily practice, patients presenting with recurrent chest pain—especially when partially responsive to nitrates—may undergo repeated coronary angiography and percutaneous coronary interventions, sometimes with uncertain clinical benefit (4).

The pathophysiology of VSA involves vascular smooth muscle hyperreactivity, autonomic dysfunction, and endothelial impairment; however, its clinical expression is highly variable, ranging from stable angina to life-threatening arrhythmias or sudden cardiac death. Diagnostic uncertainty can lead to repetitive testing and overtreatment, especially in cases where vasospasm is not recognized early in the disease course (5).

In this case report, we present the complex clinical course of a young man with refractory vasospastic angina who underwent 15 coronary angiograms and 9 stent implantations over 9 years. Despite extensive pharmacologic therapy, invasive physiological testing, and even surgical cardiac denervation, his symptoms persisted. This case highlights the challenges inherent in managing refractory vasospasm, illustrates the potential for procedural overuse, and underscores the importance of considering neuropsychological factors in persistent angina syndromes.

## Case presentation

A 42-year-old Caucasian man with a history of active smoking (ceased in 2017) and hypercholesterolemia initially presented in October 2014 with a non-ST elevation myocardial infarction (NSTEMI). Coronary angiography revealed a culprit lesion in the marginal branch of the left circumflex artery, which was treated with percutaneous coronary intervention and bare-metal stent implantation.

In September 2016, the patient experienced recurrent chest pain, prompting stenting of the mid-segment of the left anterior descending artery (LAD) with a drug-eluting stent (DES). Due to persistent symptoms, three additional DESs were implanted in the right coronary artery (RCA) in January 2017. Since lesions were identified after nitrate injection, they were interpreted as common atherosclerotic lesions and were therefore treated with stent implantation. However, recurrent chest discomfort led to further evaluation in August 2017, which revealed patent stents in all three coronary arteries.

The patient continued to experience frequent episodes of rest-related chest pain that were highly responsive to nitrates and occurred almost daily. Follow-up coronary angiographies performed in May 2018 and April 2019 did not reveal new significant lesions, and no further interventions were undertaken. However, angiographic evaluations in December 2019 and April 2020 showed progression of disease in the RCA, leading to the implantation of three DESs and one additional DES, respectively. Given the persistence of symptoms in the absence of clearly obstructive lesions and the suspicion of RCA spam, a Methergin (ergonovine) provocation test was performed in May 2020, which yielded a positive response in the distal RCA. Ergonovine and acetylcholine tests both provoke coronary vasospasm but target different receptors, causing different spasm patterns: Ergonovine acts on serotonin receptors and tend to cause focal/proximal spasms, while acetylcholine acts on muscarinic receptors and leads to more diffuse or distal spasms. Acetylcholine testing is potentially more sensitive in women and in non-CAD patients.

A subsequent coronary angiogram performed in August 2020 did not reveal any new significant stenosis.

**Figure 1:**
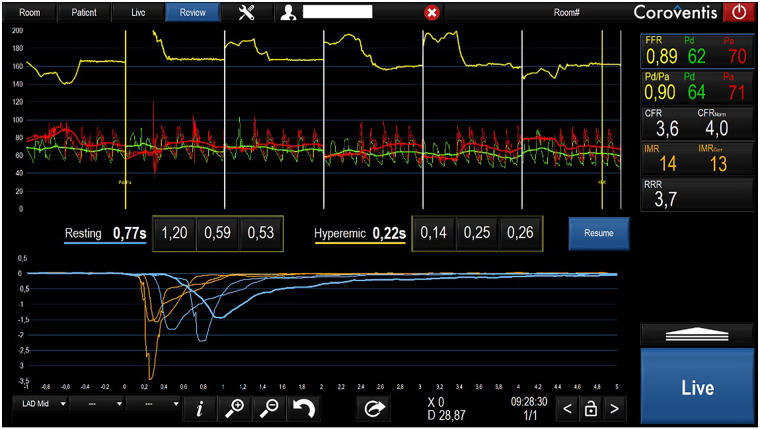
Invasive microvascular function evaluation.

In March 2021, invasive assessment of coronary microvascular function revealed a normal index of microcirculatory resistance and preserved coronary flow reserve (CFR), effectively ruling out microvascular dysfunction (Figure 1).

**Figure 2:**
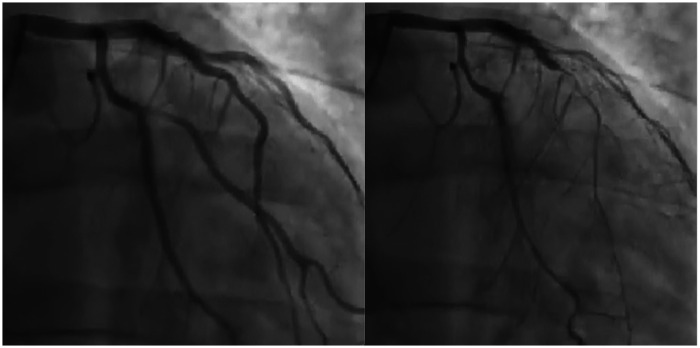
Left anterior descending artery injection before (left panel) and after (right panel) acetylcholine infusion.

Despite maximal medical therapy, the patient continued to report disabling chest pain. Initial antiplatelet therapy consisted of aspirin in combination with clopidogrel and was later switched to ticagrelor, given evidence that ticagrelor exhibits more positive effects on endothelial dysfunction than other antiplatelets. These effects are linked to its ability to raise adenosine levels, leading to activation of NO synthase, reduced inflammation, and improved vascular function. In December 2021, intracoronary acetylcholine testing provoked severe, diffuse spasm of the LAD, which resolved after nitrate administration (Supplementary Video 1 and Figure 2), confirming a diagnosis of epicardial vasospasm. The patient described pain during the test, although his ECG showed no significant changes.

Emergency department visits became increasingly frequent, although repeated ECGs and high-sensitivity troponin assays consistently remained negative. Given the severity and chronicity of the symptoms, the patient voluntarily sought evaluation at a surgical center, where a denervation intervention was proposed. At that time, however, the indication for surgical intervention was considered questionable at our center.

**Figure 3:**
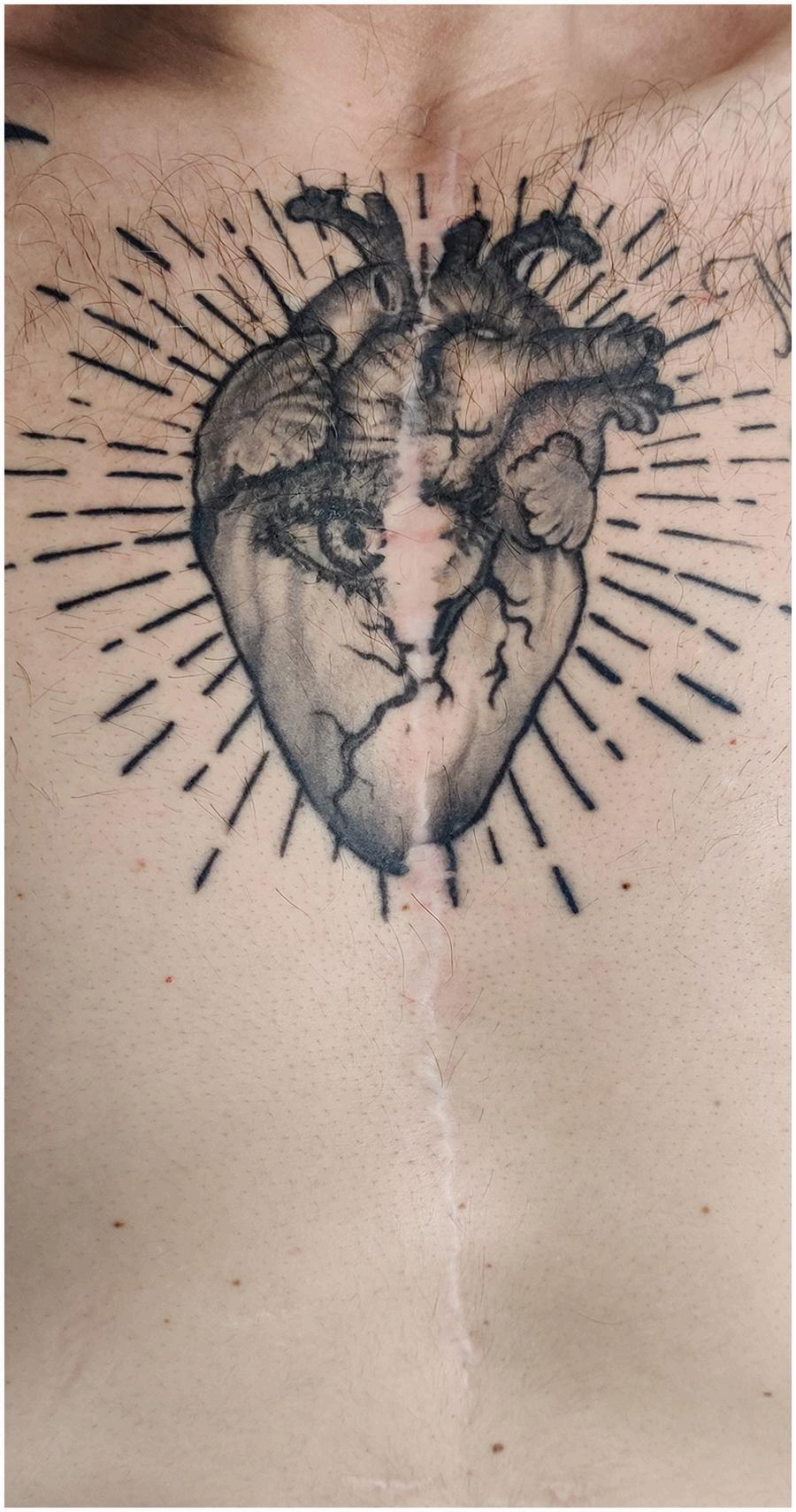
Patient's chest: Thoracotomy scar and tattoo immortalizing the cardiovascular history of the patient.

In April 2022, the patient underwent surgical cardiac denervation via cardiac autotransplantation under extracorporeal circulation. In a symbolic gesture, he later inscribed a chest tattoo commemorating his disease and its treatment (Figure 3).

Unfortunately, his symptoms recurred within weeks of surgery. Repeat angiography in May and September 2022 confirmed patent stents without new obstructive lesions; however, acetylcholine testing remained strongly positive (Supplementary Videos 1,3). In October 2022, a subsequent acetylcholine test was performed under a bilateral stellate ganglion block (using ropivacaine 0.5% and dexamethasone), yet it still induced significant vasospasm (Supplementary Video 4). These findings confirmed that the vasospasm was not mediated by the autonomic nervous system but rather driven by persistent endothelial dysfunction.

**Figure 4:**
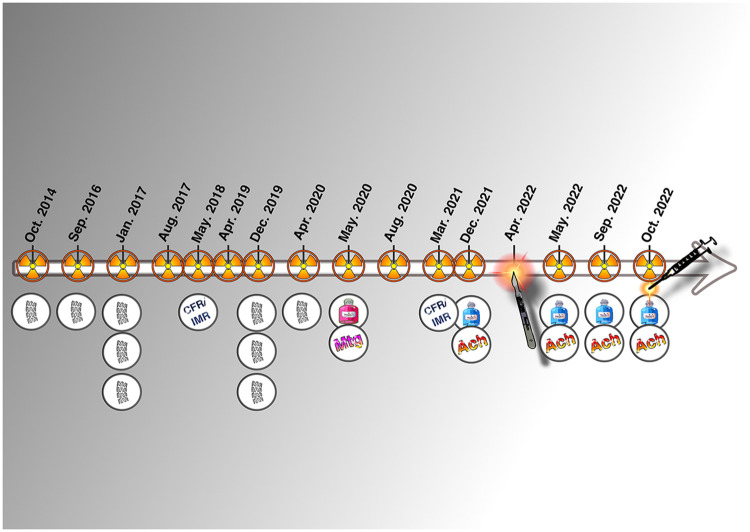
Graphical timeline of the invasive history of the patient.

Except for his first presentation as a NSTEMI, all other hospital admissions were for angina without elevation of cardiac markers. Over a 9-year period, the patient underwent a total of 15 coronary angiograms and received 9 coronary stents (Figure 4). Due to continued symptoms despite optimal medical therapy and multiple invasive interventions, the patient explored extreme options, including euthanasia, and was referred to another center for pre-transplant evaluation. At present, our team is not considering the indication of transplantation, especially since we have serious doubts regarding compliance of the patient. Strict compliance with an appropriate treatment, combined with a lifestyle correction and cardiac revalidation, appears sufficient to improve the condition of this patient.

## Discussion

VSA is a complex and underdiagnosed condition characterized by transient vasoconstriction of the epicardial or microvascular coronary arteries, often occurring in the absence of significant obstructive coronary artery disease. Its clinical presentation ranges from mild rest angina to life-threatening arrhythmias and sudden cardiac death. In patients with overlapping features of atherosclerosis and coronary spasm, as in our case, diagnostic uncertainty can lead to repeated invasive evaluations and interventions, sometimes with limited clinical benefit.

Our patient's clinical course—marked by 15 coronary angiograms, 9 stent implantations, and ultimately surgical cardiac denervation over a 9-year period—illustrates the potential consequences of attributing recurrent chest pain to fixed coronary lesions rather than to dynamic vasomotor dysfunction. While early angiographic findings revealed obstructive disease that justified the initial interventions, the persistence of symptoms despite patent stents and normal troponins should have prompted earlier consideration of vasospasm as the predominant underlying mechanism.

This case emphasizes the importance of functional coronary testing, including provocative testing with acetylcholine or ergonovine, which ultimately confirmed the presence of epicardial vasospasm. Intriguingly, invasive assessment of the coronary microvasculature showed preserved coronary flow reserve and normal microcirculatory resistance, effectively excluding microvascular angina. These findings localized the disease to large-vessel endothelial dysfunction, a key target in the pathophysiology of VSA.

Despite aggressive vasodilator therapy—including calcium channel blockers, long-acting nitrates, and statins—the patient remained symptomatic. Neuromodulatory strategies, such as stellate ganglion block and ultimately cardiac autotransplantation, were pursued based on emerging evidence that autonomic imbalance contributes to vasospasm and refractory angina (4). However, both strategies failed to suppress acetylcholine-induced spasm, suggesting that autonomic dysregulation was not the primary driver in this case, and endothelial dysfunction remained the dominant mechanism. For these reasons, although bilateral cardiac sympathetic denervation via thoracoscopic partial removal of the sympathetic trunk has been described in the literature, we did not consider this intervention appropriate at this stage.

The recurrence of symptoms after complete cardiac denervation raises important questions. From a physiological perspective, patients without cardiac autonomic innervation should not experience ischemic chest pain. However, our patient continued to report angina-like symptoms in the absence of ECG changes or biomarker elevation. This paradox suggests a possible central sensitization phenomenon—a maladaptive neuroplastic process in which the brain continues to interpret non-cardiac stimuli as ischemic pain. The emerging field of cardioneurology is beginning to explore these brain–heart interactions, particularly in patients with medically unexplained angina and refractory symptoms.

In addition, this case prompts reflection on the concept of iatrogenic burden in cardiology. The implantation of nine coronary stents, while technically justified at each time point, likely contributed to local endothelial dysfunction and may have amplified vasospastic responses. Furthermore, repeated negative angiographies did little to reassure the patient, whose healthcare trajectory became increasingly shaped by procedural escalation rather than a holistic approach to care.

From a broader perspective, the case highlights the clinical and ethical complexity of managing patients with intractable angina, especially when symptoms persist in the absence of demonstrable ischemia. While heart transplantation is not currently recommended for VSA, such patients often fall into a gray zone of therapeutic despair, as illustrated by our patient's consideration of euthanasia and subsequent referral for transplant evaluation.

## Conclusion

This case highlights the challenges of managing refractory vasospastic angina, especially when it coexists with obstructive coronary disease. Despite extensive interventions—including stenting, pharmacologic therapy, and cardiac denervation—the patient remained symptomatic due to persistent epicardial vasospasm.

The recurrence of symptoms after denervation suggests a predominant role of endothelial dysfunction and raises the possibility of central pain mechanisms. Early recognition of vasospastic angina through functional testing is therefore essential to prevent unnecessary procedures and to facilitate a more holistic, patient-centered approach.

## Patient Perspective

Over the past 9 years, I have lived with daily chest pain that disrupted every aspect of my life. Despite undergoing multiple stents, surgeries, and medications, the pain never truly disappeared.

## Data Availability

The original contributions presented in the study are included in the article/Supplementary Material; further inquiries can be directed to the corresponding author.

## Supplementary material

Supplementary Video 1 Coronary angiogram with acetylcholine test performed in December 2021.

Supplementary Video 2 Coronary angiogram with acetylcholine test performed in May 2022 1 month after cardiac denervation.

Supplementary Video 3 Coronary angiogram with acetylcholine test performed in September 2022 5 months after cardiac denervation.

Supplementary Video 4 Coronary angiogram with acetylcholine test under bilateral local stellate ganglions block performed in October 2022.
